# Marine Pollution and Advances in Biomonitoring in Cartagena Bay in the Colombian Caribbean

**DOI:** 10.3390/toxics11070631

**Published:** 2023-07-20

**Authors:** Patricia Romero-Murillo, Jorge L. Gallego, Vincent Leignel

**Affiliations:** 1 Escuela de Biología Marina, Grupo de Investigación GIBEAM, Universidad del Sinú Seccional Cartagena, Av. El Bosque Trans, 54 N° 30-453 Santillana, Cartagena de Indias 130014, Colombia; 2 Grupo de Investigaciones y Mediciones Ambientales GEMA, Facultad de Ingenierías, Universidad de Medellín, Carrera 87 N° 30–65, Medellín 050026, Colombia; jlgallego@udemedellin.edu.co; 3 Laboratoire BIOSSE, Le Mans Université, Avenue O Messiaen, 72000 Le Mans, France; vincent.leignel@univ-lemans.fr

**Keywords:** bioaccumulation, biomarker, ecosystem services, persistent organic pollutants, sediment, trace metals

## Abstract

Coastal zones sustain extensive biodiversity, support key processes for ocean dynamics, and influence the balance of the global environment. They also provide resources and services to communities, determine their culture, and are the basis for their economic growth. Cartagena Bay in the Colombian Caribbean is the place of the establishment of one of the country’s main cities, which has a great historical and tourist attraction, and it is also the location of the main commercial port and a great variety of industries. Historically, it has been affected by several environmental impacts and intense pollution. This situation has gained the attention of different researchers, so herein is presented a literature review with a systematic approach using RStudio’s bibliometrix on the presence of pollutants and the impact on biodiversity in recent decades, providing a critical analysis of the state of Cartagena Bay and its future needs to ensure its recovery and conservation. In addition, the socioeconomic dynamics related to the environmental state of Cartagena Bay are presented from the framework drivers, pressures, status, impacts, and responses (DPSIR). The update and critical understanding of the sources, fate, and effects of pollution are important not only for the knowledge of the status of this singular ecosystem but also to encourage future research and entrench evidence to support decision makers’ actions. This review highlights that several pollutants that have been detected exceeding sediment quality guidelines, like As, Cd, Hg, and PAH, are also reported to bioaccumulate and cause damage throughout the trophic levels of the coastal environment. In addition, the potential use of sentinel species and biomarkers for their monitoring is discussed. Finally, the factors that cause pollution and threaten the state of the bay continue to exert pressure and impact; thus, there is a call for the further monitoring of this ecosystem and the strengthening of policies and regulations.

## 1. Introduction

Coastal areas are fragile ecosystems, strongly modulated by anthropogenic processes, and conditioned to the complexity of dynamical exchanges with the terrestrial environment. Their structure is composed of seagrasses, coral reefs, estuaries, mangroves, and open waters, providing habitats that sustain a high biodiversity and valuable ecosystem services [1]. As their natural capital provides livelihoods, raw materials, food, goods, and assets, coastal ecosystems are key for the development and economic growth of regions [2]. However, these characteristics have led to clusters of specialized industries [3,4], as well as an increase in the coastal population and urbanization [5], originating environmental impacts. Furthermore, coastal areas and oceans receive direct discharges of wastewater, runoff, and inland waters, serving as final sinks for global pollution [6,7,8,9]. This results in the deterioration of the water quality [10,11] and the accumulation of contaminants, such as trace metals [12,13,14], pesticides [15,16], hydrocarbons [17], and persistent organic pollutants [18,19]. In addition, there are increasing reports about the impact of marine debris, plastics, microplastics [20,21,22], emerging pollutants such as personal care products [23], pharmaceuticals [24], flame retardants [25], synthetic drugs [26], and surfactants [27] found in water, sediments, and marine organisms.

Contaminants in the marine environment present a complex dynamic in the water column, suspended materials, sediments, and organisms [28]. Sediments, especially, are considered a reservoir of pollutants, as they can interact with substances of different natures, regulating their adsorption, attenuation, or accumulation [29,30,31]. In addition, biotransformation reactions may occur, leading to the partial degradation of the pollutants or to the formation of different metabolites that in some cases are more toxic or mobile than their parent compounds [32,33,34]. In this way, pollutants become bioavailable and are transferred to organisms of different trophic levels through bioaccumulation and biomagnification [35,36]. Several species have characteristics that allow them to detect the impact of disturbances in the marine environment, presenting effects at the molecular, biochemical, histological, physiological, and morphological levels [37] and even at community and ecosystem structure levels [38,39]. Therefore, strategies for the assessment of coastal pollution include two approaches: the monitoring of substances in sediment, water, and suspended material and the use of sentinel organisms, such as macroinvertebrates [40,41], foraminifers [42], algae [43,44], polychaetas [45], ascidians [46], echinoderms [47,48], bivalves [49,50,51,52], crustaceans [53,54,55,56], and fish [57,58,59], among others sensitive to environmental changes.

The conservation of coastal zones is a global priority. Each region has unique ecosystem structures and functions; thus, it is necessary to recognize the nature and magnitude of the impacts that endanger them [60]. In the Latin American and Caribbean region, the degradation of coastal ecosystems and overexploitation of natural resources is a major concern [61]. Although the implementation of the Sustainable Development Goals has mobilized government efforts, these are poorly coordinated, have little institutional capacity, and have limited resources [62,63]. Therefore, it is necessary to contribute to the knowledge on the state of these ecosystems, to recognize pollution challenges, and to contribute to the analysis of alternatives for decision makers.

Cartagena Bay, located in the north coast of Colombia, is a diversified ecological zone, including mangrove areas, seagrasses, and coral reefs [64,65,66,67]. It is also a strategic area for the development of the country and the Caribbean and is becoming an economic region that gathers industrial, tourist, international trade, and port activities [68]. However, these activities are sources of pollution and ecosystem degradation, which represent the major environmental challenges for the sustainable management of the bay [69]. This area has been historically affected by oil accidents [70,71], pesticide spills [72], mercury discharges from a chlor-alkali plant [73], ballast waters from ships [74], industrial discharges, urban wastewater, and solid wastes [75,76,77,78]. Consequently, pollution assessments in the bay and biomonitoring in different species have revealed bioaccumulation and the negative effects of environmental degradation [13,59]. Several publications have addressed the occurrence of pollutants, biomonitoring approaches, and environmental risks resulting from the continued contamination that has been generated in this area, which is one of the most studied ecosystems in Colombia and the Caribbean [79,80,81,82,83]. Therefore, a need arises to analyze the reported substances, concentrations, risks, and effects on some species, with the objective of providing a synthesis of the status and gaps in the study of Cartagena Bay. This review aims to consolidate the scientific contributions of the last few decades on the status of this representative ecosystem of the Colombian Caribbean. Finally, we outline monitoring approaches as well as the need for protection policies and actions.

## 2. Systematic Literature Exploration 

This review was developed through a literature exploration with a systematic approach using RStudio’s bibliometrix work package [84]. To select the relevant publications from Scopus, the search descriptors were defined as follows: “Caribbean, Colombia” OR “Cartagena bay” AND “bioaccumulation” OR “biomarker” OR “ecosystem services” OR “marine organism” OR “persistent organic pollutants” OR “sediment” OR “trace metals”, from the following questions: (1) Which are the matrices mainly studied? (2) Which are the most relevant pollutants? (3) What are the information gaps? The search yielded a total of 38 article-type documents, including titles, keywords, abstracts, and publications in English, of which those related to Cartagena (Spain) were excluded from the analysis, resulting in the analysis of 22 articles. The analysis included information generated between the years 1988 and 2022.

## 3. General Description of Cartagena Bay

**Figure 1:**
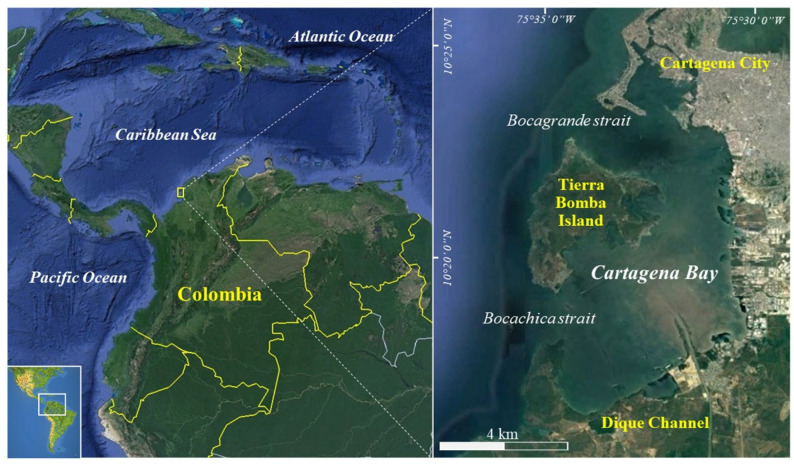
General location of the Colombian Caribbean and Cartagena Bay, respectively.

Cartagena Bay is located on the northern coast of Colombia (10°17′54.29″ N, 75°35′08.91″ W to 10°23′49.88″ N, 75°33′54.63″ W) (Figure 1). The coastline is delimited by the city of Cartagena, with mainly tourist and residential areas to the north, followed by the port area and the industrial zone to the south, including the refinery, cement plant, and agrochemical, food, and polymer industries [69,75]. The areas surrounding the bay correspond to the tropical dry forest life zone, most of which are urbanized and heavily intervened [68]. These areas make up a complex water network with slightly flat and intermittent drainages, lagoons, and mangroves, many of them intervened with artificial canals [85,86]. The Tierrabomba Island delimits the bay to the west, forming a water surface area of 84 km^2^ with an average depth of 16 m, and it is connected with the Caribbean Sea through the Bocagrande strait in the northwest and the Bocachica strait to the southwest [87].

The average annual rainfall is 1052 mm, with a temporal distribution in two dry periods, December to April and July to August, and two rainy periods, May to June and September to November. The latter makes up 55% of the total precipitation [88]. The average temperature is 28 °C, the water annual temperature oscillates between 25 and 30 °C, and the winds are predominantly north-easterly, with an average velocity of 8 m/s during the dry season and weaker during the wet season; in addition, their effect on the distribution of currents is greater than that of the swell [75,76]. Tidal dynamics are characterized as mixed and mainly diurnal [89] with mean sea level fluctuations between 0.43 and 0.55 m of tide amplitude [90].

The hydrodynamics of the bay are influenced by the Dique Channel, built during the seventeenth century to connect the region with the fluvial transportation network through the Magdalena River [91]. The bay receives a 55 to 300 m^3^s^−1^ flow in the southern area from the channel during dry and wet periods, respectively [92], acquiring an estuarine behavior [76,77]. The Dique Channel has an important impact on the environmental quality of the bay due to the flow of freshwater and the transport of pollutants, nutrients, and sediments [77,87,93]. The sediment load to the bay has been estimated to be 2.6 and 1.3 Mt y^−1^ during the wet and dry periods, respectively [94], which are evenly distributed until reaching the coral substrates and accumulated in the area due to the shallow depth of Bocachica [95]. In general, these geographical characteristics of Cartagena Bay determine the dynamics of local communities, the tourism industry, and economic development activities, which significantly influence the environmental changes, impact of pollution, and adaptation to climate change.

## 4. Environmental and Socioeconomic Dynamics in Cartagena Bay

The socioecological processes surrounding Cartagena Bay reflect a multifaceted complexity, with significant implications for its conservation status and environmental health [82,96,97]. In general, the coastal and marine ecosystem services determine the socioeconomic dynamics of a region and are crucial for vulnerable communities, whose dependence increases strongly in climate change scenarios [2,98]. Hence, there is growing concern that ecosystem protection and restoration strategies are still slow or ineffective in the Caribbean region [99,100]. In the case of Cartagena Bay, capital, infrastructure, technology, and governance constraints need to be addressed to strengthen the sustainable management of ecosystems and their pollution problems [69]. In this context, a general overview of drivers, pressures, status, impacts, and responses (DPSIR) is presented in Table 1, in order to describe the socioeconomic and environmental trends influencing the pollution status of Cartagena Bay. The DPSIR framework was developed with available information in the literature of Cartagena Bay for selected indicators for each component [101,102].

Drivers are related to the major forces affecting the bay from both natural and socioeconomic dimensions. Since the 1960s, Cartagena Bay has been highly modified because of the economic development focused on the petrochemical industry, the international trade, and the increase in fishing and tourist activities [130]. The city has one of the highest urbanization rates in the region [65]. In the last few decades, the population of Cartagena de Indias increased from 895,400 inhabitants in 2005 to 1,055,035 inhabitants in 2022 [103]. The city has been declared on the World Heritage List by UNESCO since 1984 [131]. Nowadays, it has become also one of the most important Colombian tourist centers with a 73% increase in cruise ship visits from 2008 to 2018 and 15% increase in international air arrivals from 2018 to 2022; in addition, 24% of the maritime exchange in the Caribbean region is transmitted through Cartagena Bay [132,133]. In terms of drivers of natural phenomena, the forces of global environmental change are considered. The city ranks among the top ten in the country with a high-risk rating for the effects of climate change, including increased temperatures, water scarcity, a risk of flooding, an increased intensity of extreme events, and the acidification of the sea [121].

The second component of the DPSIR analysis is the pressure exerted by driving forces. Due to the demographic, economic, and natural forces, several pressures are identified in Cartagena Bay. Although tourism occupies an important position, the economy continues to grow with a high dependence on the industry of the primary sector, increasing demands for water, raw materials, and the generation of industrial solid wastes and wastewater [75,134]. For instance, by the year 2021 the local authorities reported permissions for the use of 14,647.4 L/s of surface water and 7.4 L/s of groundwater, in addition to the authorization of discharges for 15,388.8 L/s of wastewater, issued to different industries like oil and gas industries, chemical producers, plastics industries, tanneries, cement industries, ports, thermoelectric industries, mining, and pesticide production [75]. In addition, domestic solid waste generation and wastewater discharges are common in peripheric areas where marginal conditions persist [96].

The third component, status, refers to the current environmental condition under the synergistic interaction of drives and pressures. The state of Cartagena Bay has changed during the last few decades. Fragile ecosystems have been transformed or reduced because of the intensification of industrial and infrastructure development and urban and tourist activities, which threaten mangroves, coasts, and conservation areas [64,68,97,108,113]. Specifically, the continued presence of contaminants in sediments, beaches, water, marine organisms, and birds [59,80,116,118,125,135,136,137,138] has been reported, which evidence the deterioration of the bay and the threat to the health of the communities and their livelihoods.

The changes in the environmental status of the bay over time have manifested diverse impacts on ecosystems, biodiversity, and the surrounding population. In relation to the component impact, studies have shown alterations in the physicochemical and microbiological quality of the water [126,128,135], changes in fishery species, a risk of economic losses [119,120], the altered health of marine organisms [59,122,125], and exposure to marine pollutants in vulnerable communities [81,82]. The progressive evidence of these significant changes and the continuing environmental deterioration of the bay have wider implications that emphasize the urgent need to adopt pollution control and mitigation actions, preserve natural habitats, and preserve the health of both the marine environment and the affected populations.

Finally, the DPSIR component called responses identifies management strategies and policies applied to mitigate coastal pollution that involve institutional agreements, research programs, and education campaigns. These include the program Basin Sea Interactions with Communities (BASIC) between the years 2014 and 2021, which aimed to contribute to the environmental governance of Cartagena Bay through scientific and institutional alliances and achieved scientific analysis of the state of the bay with environmental policymakers, coastal communities, and decision makers, leading to various political impacts, including increased studies, monitoring tools, and mitigation measures for pollution, as well as the establishment of an Intersectoral Environmental Committee and enhanced regulatory control over industrial discharges [129]. In addition, in 2014 the mayor’s office of Cartagena developed a climate change adaptation management plan, prioritizing strategic ecosystems and vulnerable communities in the identification of hazards and vulnerability analysis for the formulation of adaptation actions [121]. With regard to the impact of industrial activities, in 2021 the environmental authorities created an information system on the most relevant aspects of the state of the bay to support decision making regarding new authorizations for projects being developed in the area [75]. Despite these efforts, it must be recognized that the worrying trends in Cartagena Bay require a thorough understanding of the dynamics, effects, and future scenarios of marine pollution to strengthen effective strategies for its environmental recovery.

## 5. Diversity of Domestic and Industrial Pollution in Cartagena Bay

**Figure 2:**
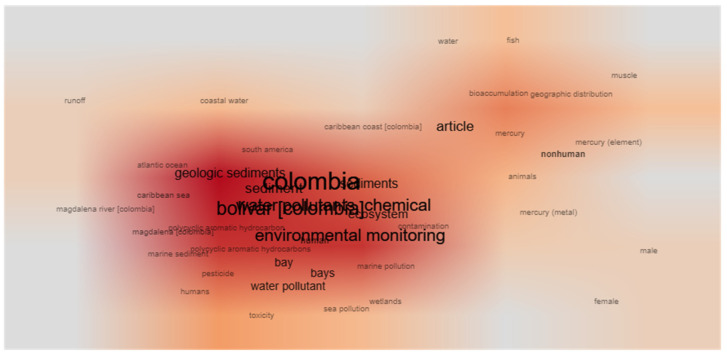
Keyword co-occurrence network of selected studies from Scopus.

Multiple anthropogenic activities and the entry of inland waters through the Dique Channel release a variety of pollution into Cartagena Bay. However, as described below, studies have been mainly concentrated on the monitoring of metals, polycyclic aromatic hydrocarbons (PAHs), pesticides, persistent organic pollutants, and, more recently, plastics and some emerging pollutants. According to the bibliometric analyses (Figure 2), the studies focused on analyzing contaminants in sediment samples, and, in a lesser proportion, in the water column. Therefore, the evaluation of sediment quality has been the main objective of recent studies in the bay. However, Colombian legislation lacks a regulation or definition of specific standards for sediment monitoring. In this sense, the use of sediment quality guidelines (SQGs) has become a meaningful tool to determine the toxicological relevance of pollutants associated with marine sediments. The most commonly used SQGs are the threshold effect level (TEL), which is defined as the level below which adverse biological effects will rarely occur, and the probable effect level (PEL), which represents the concentration above which adverse effects are frequently expected [139]. In general, the results of the SQGs can be interpreted as follows: pollutant concentrations below the TEL are not associated with adverse biological effects; those concentrations between the TEL and PEL may occasionally be associated with toxic biological effects; and values higher than the PEL are linked with adverse biological consequences [30].

In the assessment of trace metals, in addition to the biological effects criteria, there are indexes based on the comparison of the total concentration of metals in sediments with the background concentrations. The most common is the geoaccumulation index (*I_geo_*), developed initially for the assessment of the sediment quality of rivers [140]. It is calculated as *I_geo_* = Log_2_(C_n_/1.5 GB), where C_n_ is the concentration of an individual metal and GB is the value of the geochemical background, resulting in values from *I_geo_* < 0, which are considered unpolluted, to *I_geo_* > 5, which are considered extremely highly polluted [141]. The geochemical background corresponds to the natural metal concentrations prior to human influence, serving as a reference point for assessing the extent of anthropogenic-induced changes. These values should be measured with sediment cores or otherwise selected from the literature, taking care to be consistent with local conditions [142]. This approach is used by the *I_geo_* and other indices, such as the contamination factor, enrichment factor, pollution load index, metal pollution index, and Nemerow pollution index [143].

### 5.1. Pollution by Hydrocarbons

Hydrocarbons are natural compounds that can be synthesized by organisms and found in fossil fuels, whose alteration in the environment has been caused by anthropogenic activities, such as combustion and transformation processes for the manufacture of diverse products [144]. However, the greatest concern lies with polycyclic aromatic hydrocarbons (PAHs), which may have greater toxicological impacts. There are several classes of PAHs based on the number of benzene rings they contain; as the number of rings increases, they tend to aggregate and adhere to the marine sediments due to their stable hydrophobic structures [145]. 

As discussed in the DPSIR analysis, the refinery, maritime traffic, and urban runoff are the main sources of hydrocarbons in Cartagena Bay [75,83]. One of the first reports in the bay made in the 1980s recorded concentrations of total hydrocarbons dispersed in surface water between 10 and 20 µg/L [71]. These results are similar to those reported by a monitoring conducted in 2019 in the surface waters of the industrial zone of the bay (4.1–18.8 µg/L) [146]; that is, similar levels of dispersed hydrocarbon contamination were observed in Cartagena Bay 30 years later. Regarding sediment monitoring, total hydrocarbons were monitored in surface sediments and were found to range from 2.2 to 1415 µg g^−1^ during the years 1996 and 1997 [83]. A study evaluated the presence of PAHs in sediment cores ranging from 148.3 to 1603.6 ng g^−1^ ΣPAHs. In addition, the chronological approach allowed the deeper layer (59–65 cm) to be associated to the years 1965 to 1970; the middle (35–59 cm) to the years 1970 to 1987; and the uppermost (0–35 cm) to the years 1987 to 2010, with distribution patterns found for the high PAHs like dibenzo[a,h]anthracene, fluorene, and benzo[a]pyrene, especially at 40 cm depths, whereas those of lower molecular weights, like naphthalene and phenanthrene, were recorded in the surface layers [147]. A subsequent study in 2003–2004 reported ΣPAHs of 1330, 1740, and 3210 ng g^−1^ on the sediment fractions of 20, 30, and 40 mesh particle sizes, respectively, and average concentrations from 13.8 to 526.0 ng g^−1^ for individual the PAHs fluorene, phenanthrene, anthracene, fluoranthene, chrysene, pyrene, benzo[a]anthracene, benzo[b]flurantene, benzo[a]pyrene, dibenzo[a,h]anthracene, indeno(1,2,3,cd)pyrene, and benzo[g,h,i]perylene [123]. The study found that Cartagena Bay had the highest degree of PAH contamination compared to other coastal water bodies in the Colombia Caribbean (Totumo marsh and Caimanera marsh). A more recent study performed in 12 monitoring stations reported ΣPAHs from 16.6 to 571 ng g^−1^ in sediments from the bay [148].

Table 2 presents the comparison between both studies and the reference site Santa Marta Bay, also located in the Colombian Caribbean, which is recognized as a tourist and industrial zone and especially as an area of influence of the coal ports; however, the reports of PAHs are still higher in Cartagena Bay.

### 5.2. Pollution by Pesticides and Persistent Organic Compounds

The presence of pesticides and persistent organic compounds in Cartagena Bay has been associated with the activities and accidents of the chemical industries in the area [72,136]. However, it is also important to consider the agricultural activities across the country, particularly in areas where excessive pesticide use has been reported [149,150], affecting the rivers that flow through the main watershed of the country until they reach the bay through the Dique Channel [94,112]. Colombia intensified the use of persistent pesticides during the 1970s, including aldrin, dieldrin, endrin, chlordane, heptachlor, hexachlorobenzene, mirex, toxaphene, and DDT [151], after their prohibition was replaced by organophosphates, carbamates, pyrethroids, neonicotinoids, and benzimidazoles, among other currently used pesticides of great importance for agriculture in the country, registering an average annual use of 42,887 tons [152]. Colombia adopted the Stockholm Convention and is advancing in the elimination and remediation of affected areas; however, the footprint of the persistent organic compounds is still registered in soils, rivers, and coasts [116,153].

Most of the studies in Cartagena Bay have monitored organochlorine pesticides (OCPs). In the year 2009, sediment cores were extracted from a depth of 65 cm and the total OCPs aldrin, dieldrin, heptachlor and its epoxide, hexachlorocyclohexanes, DDT and its isomers DDEs, and DDD presented a maximum record of 150 ng/g of total OCPs at depths between 30 and 40 cm, which, according to the chronological analysis, corresponds with the 1980s and 1990s when their prohibition was just being regulated [147]. In the same study, hexachlorocyclohexanes and endosulphans were found in surface sediments (0–20 cm) in concentrations of 10 to 30 ng/g. Between 1997 and 2001, a study assessed chlorinated aromatic compounds in sea surface water and the pesticide concentrations detected in the sampling location on the coast of Cartagena de Indias were 2.5 ng/L of chlorinated benzenes, 6.1 ng/L of hexachloro cyclohexanes, 4.1 ng/L of chlordane compounds, 3.7 ng/L of other cyclodiene pesticides, 10 ng/L of DDT-related compounds, and 75.5 ng/L of PCBs [18]. The pesticides thiocarbamates, bromacil, triazines, organochlorines, and organophosphorus were reported in sediments of Cartagena Bay in 2015 ranging from 0.83 to 33.67 ng/g, and polychlorinated biphenyls (PCBs) were also reported, ranging from 0.06 to 19.58 ng/g [116]. In 2017 and 2018, total PCBs were reported in sediment samples (n = 12) in concentrations of 15.2 ng/g to 18.59 ng/g (PCB 138 was the most frequent detected); DDT metabolites DDD and DDE ranged from 0.069 to 0.61 ng/g; chlorpyrifos were detected from 0.42 to 1.33 ng/g; and deltamethrin was found in two sampling sites in concentrations of 1.87 ng/g and 10.26 ng/g, respectively [148].

### 5.3. Pollution by Trace Metals

Trace metals are relevant pollutants in seawater and sediments. They occur naturally and may be increased in the marine environment by anthropogenic action originating from many industrial, tourist, and domestic activities; specifically, in Cartagena Bay they are related to cargo ports, tourist boats, the metal-mechanic industry, welding, the old chlor-alkali plant, mining, cement, and oil refineries, as well as industrial and domestic pollution in the interior of the country that contaminates the Magdalena River and the Canal del Dique [64,80,93]. In Cartagena Bay, mercury (Hg) has been reported at concentrations of 18.76 µg/g in sediment at a 60 cm depth [147]. In 1996, analyses of sediment in the bay showed concentrations of 0.094 to 10.293 μg/g Hg [69]. Then, in 2006 it was found in an average of 0.18 ± 0.01 μg/g Hg [154]. In 2014–2015, analyses were carried out for samples from the bay, Dique Channel, and Barú Island (located to the south on the outer coast of the bay), showing concentrations of 0.131, 0.091, and 0.029 μg/g Hg, respectively; additionally, methyl mercury ranged from 0.0014 to 0.0245 μg/g, which indicated that 2–20% of the total mercury was bioavailable [135]. 

Other metals in sediment in 2014 yielded concentrations of 0.36 µg/g Cd, 24.4 µg/g Ni, 6.7 µg/g Pb, and 199 μg/g Zn [155]. Additionally, there were high concentrations of Cd in the Dique Channel with respect to the bay with 1.267 ± 0.779 μg/g and variations related to the climatic season, between 511 ± 208 μg/g in the rainy season, and 0.060 ± 0.088 μg/g in the dry season; subsequently, from sediment analysis at 12 points in Cartagena Bay, concentrations of various metals were determined (As, Ba, Be, Bi, Ce, Co, Cr, Cs, Cu, Cd, Dy, Er, Eu, Ga, Gd, Ge, Hf, Ho, Li, La, Lu, Nb, Nd, Ni, Pb, Pr, Rb, Sb, Sc, Sm, Sn, Se, Sr, Ta, Tb, Th, Tl, Tm, U, V, Y, Yb, Zr) [80]. The results indicated that most of the evaluated stations are considered moderately to highly contaminated according to the geoaccumulation index (*I_geo_*) and that the climatic season can affect the fluctuation of metal concentrations. Finally, researchers recommend special attention to As, Cd, Pb and especially Hg, which exceeded the Effects Range Medium. Table 3 sumarizes the range of metal concentrations in sediments.

### 5.4. Pollution by Microplastics and Emerging Pollutants

Microplastics are pollutants of growing concern, requiring more research to better understand their evolution in the bay and their impact on organisms. Until now, the investigations carried out indicate that Cartagena is considered a hotspot for the production of microplastics [156]. Plastics have been detected on tourist beaches in the city where pellet-type microplastics were evaluated in surface sand and it was identified that most of the pellets found had a low degree of deterioration, mainly polyethylene, followed by secondary polypropylene, possibly from the urban center and especially from short-term residents as well as contributions from nearby rivers [118,157]. It was determined that these microplastics accumulate and transport toxic elements such as metals (Ba, Ce, Cr, Ni, Pb, Rb, Sr, Zr) and can be toxicologically dangerous [157]. Also, the presence of these microplastics is related to the high production of wastewater and solid waste. In addition, organophosphate flame retardants tris (2-ethylhexyl) phosphate (TEHP), tris-ortho-tolyl phosphate (ToTP), and 2 ethylhexyl diphenyl phosphate (EHDPP) were detected in ranges of 0.11 to 11.17 ng/g, 0.68 to 1.12 ng/g, and 0.25 to 0.29 ng/g, respectively [148]. The same study monitored UV filter 4-methylbenzylidene camphor (4MBC) ranging 0.32 to 52.83 ng/g in 33.3% of the sampling sites, while homosalate was below the limit of 0.022 ng/g for all the samples; in addition, the occurrence of the fragrances celestolide (0.07–3.75 ng/g), tetramethyl acetyloctahydronaphthalenes (OTNE) (1.06–45.37 ng/g), tonalide (0.24–2.25 ng/g), and galaxolide (1.56–19.06ng/g) was observed. Polybrominated diphenyl ethers (PBDEs) were analyzed in sediments from 10 sampling sites in Cartagena Bay, ranging from 0.02 to 0.40 ng/g [116]. Despite the reports of emerging pollutants, there is a lack knowledge of the dynamics, distribution, accumulation, and potential negative effects on marine organisms in Cartagena Bay [18,118,156,157].

## 6. Biomonitoring of Pollutants and Impacts on Marine Animals in Cartagena Bay 

**Figure 3:**
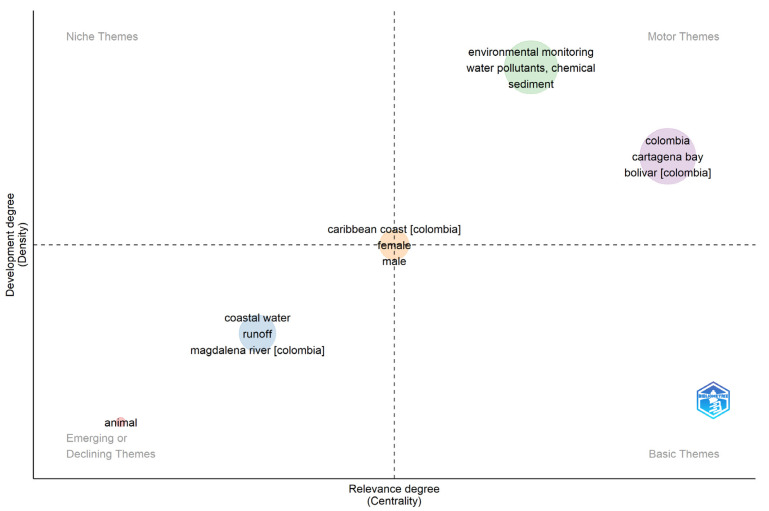
Relevance and identification of emerging themes.

In Cartagena Bay, investigations have been carried out to determine the impact of contamination on some groups of organisms like crustaceans, fish, and oysters in relation to the registered pollutants. In general, after the research on the occurrence of pollutants in sediments and water, the bioaccumulation in various organisms is the main approach of the research found on monitoring of Cartagena Bay, and few studies are related to specific biomarkers that determine the effect of pollutants on the marine organisms in the ecosystem (Figure 3).

**Figure 4:**
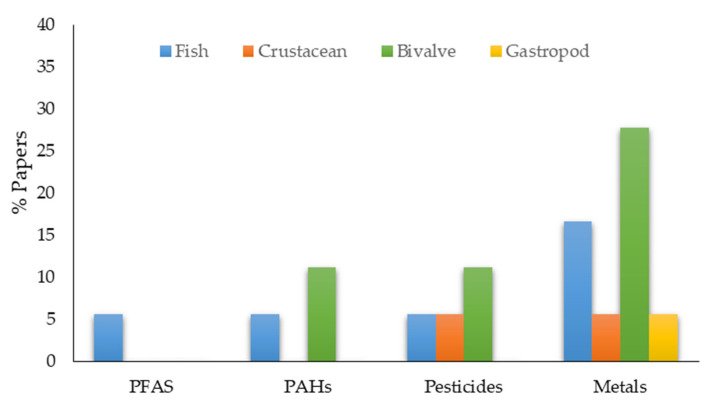
Distribution of publications according to the type of pollutants and the groups of organisms analyzed.

In detail, studies of pollutant concentrations in organisms with different trophic levels from primary to secondary consumers (Table 4) are mainly carried out with bivalves and trace metals are the most frequent target pollutants (Figure 4).

### 6.1. Biomonitoring of Organic Pollutants, Per- and Polyfluoroalkyl Substances (PFASs), Polycyclic Aromatic Hydrocarbons (PAHs), and Pesticides

The research carried out on PFOS, PFOA, PFHxS, and PFOSA in fish for human consumption in populations surrounding the coastal area has demonstrated the presence in Cartagena Bay of substances that have been shown to cause alterations in the neuroendocrine system, in particular, PFOA and PFHxS [163] (Table 5). The research conclusions highlight the need to develop programs that reduce exposure to these pollutants. The exploration and search for information on PAHs in the bay has focused on the analysis of the detritivore fish *M. incilis* and the mangrove oyster *C. rhizophora*. Similar to that recorded in sediment, high concentrations of PAHs were recorded in Cartagena Bay in the bile of *M. incilis* fish compared to local reference sites Totumo marsh and Caimanera marsh [123]. *C. rhizophora* has been identified as being sensitive to temporal changes in PAH concentrations, with higher concentrations of phenanthrene at all locations in the dry season; in addition, during the rainy season fluorene and anthracene had the highest concentrations, followed by chrysene and to a lesser extent pyrene, benzo[a]anthracene, benzo[ghi]pyrene, and indeno [1,2,3-cd]pyrene, all of which have carcinogenic potential [164] (Table 5). *Penaeus vannaemei* shrimp have been used as a bioindicator of pesticide concentrations, and their results were below the maximum limit allowed for aquatic species [124]. Also, low levels of pesticides in the muscle tissue of *M. incilis* fish has been reported [136]. Finally, findings in *Saccostrea* sp. [50] with pesticide concentrations below the detection limit are consistent with this work conducted on *M. incilis* and *P. vannamei* (Table 5).

### 6.2. Biomonitoring of Metals

Research with the aim of biomonitoring metals has been conducted on fish, crustaceans, and bivalves, the latter of which had the largest number of studies in Cartagena Bay (Figure 4). A study carried out on the fish mullet (*Mugil incilis*), catfish (*Bagre marinus, Cathorops mapale*), snapper (*Lutjanus* cf. *griseus*), and amberjack tuna (*Seriola rivoliana*) indicated low metal concentrations (zinc > nickel > lead > cadmium) compared to the maximum allowable concentrations according to international standards; and the presence of metals in fish coincides with the degree of industrialization compared to other regions [155]. A 1996 study compared mercury concentrations between fish with different diets, the detritivorous *Mugil incilis* and the omnivorous *Eugerres plumieri*, revealing higher concentrations in the omnivorous trophic level, above the international guideline of 0.5 µg/g [73]. Additionally, a subsequent study conducted in 2006 included 18 fish species and reported higher total mercury concentrations in carnivorous species, followed by omnivorous and detritivorous species, without exceeding the reference value of 0.5 ug/g [59] (Table 6). They suggested that human consumption of carnivorous fish should be avoided in vulnerable groups such as pregnant women. A study was also carried out on crabs (*Callinectes sapidus* and *C. bocourti*), which found high mercury concentrations in the individuals collected near the industrial infrastructures [125]. The authors recommend that, even if the concentrations do not exceed the risk level determined by the USEPA, fishermen who generally consume this type of seafood should be monitored.

In Cartagena Bay, research has been also carried out on oysters to identify potential species that can be used as sentinels for ecotoxicological biomonitoring. The *Crassotrea rhizophorae* and *Isognomon alatus* had high potential to be used in quantitative biomonitoring after measurements in the Colombian Caribbean [165]. In addition, the *C. rhizophorae* has been used to assess the bioaccumulation of As, Cd, Fe, and Pb in the mangrove ecosystem, showing moderate to extremely high metal concentrations according to seasons and sites [164]. A study in the oysters *C. rhizophorae* and *Saccostrea* sp. has recorded Cd in concentrations above the permitted limit of 1.0 µg/g for the Colombian Ministry of Health and Social Protection (Resolution 122 of 26 January 2012) [50]. In general, the research on bivalves [51,164,165], crustaceans [125], and fish [59,73,155] has determined that concentrations of Cd and Hg registered in Cartagena may have potential effects on aquatic life and with reference to the hazard index (HI) and food guidelines could become a threat for human health because of the importance of these species in the diet [59].

### 6.3. Biomarkers and Effects of Pollutants in Marine Organisms

Regarding the use of biomarkers to determine the effects of pollutants in Cartagena Bay, most of the studies have reported bioaccumulation in organism tissues but less has been reported about negative effects. However, some physiological, morphological, and molecular biomarkers have been used with marine organisms from the bay as sentinels (Table 7). The research on the *C. rhizophorae* and *Saccostrea* sp. has evaluated the incidence of metals and pesticides through biochemical markers, such as metallothioneins and acetylcholinesterase activity, finding a reduced content of proteins correlated to tissue and metal sediment concentrations [50]. In addition, there is evidence of the utilization of other biomarkers from molecular to morphological levels that demonstrate the sensitivity of different species as sentinels of specific pollutants, but it is less specific. The species *Mugil incilis* has been studied according to its condition factor to compare different sites [163], and together with 17 other species, were associated with total mercury content, finding a correlation with their morphometric index [59]. In addition, the species *M. incilis* was used as a model for molecular biomarkers of gene expression obtained via the RNAseq technique [58]. Table 7 summarizes species, biomarkers, and effects reported in Cartagena Bay as an application of the biomonitoring approach for specific pollutants or comparison of the general conditions of different areas.

## 7. Conclusions

This review summarized the pollution status of Cartagena Bay, exposed the main pollutants, and indicated the sentinel species used in marine ecotoxicology in this region of Colombia. The levels of some pollutants reported in studies over the last few decades exceeded the sediment quality guidelines at levels with the potential to induce negative effects on biodiversity and disturb the ecosystem services. Institutional responses have partially addressed some of the causes, mainly controls on industries, the treatment of municipal wastewater, and the study of the influence of the Dique Channel. However, other macro factors continue to affect the bay, such as the weaknesses in the authorities’ controls and the delay in land-use planning policies. The future of Cartagena and the Colombian Caribbean face great environmental challenges, and global change will exacerbate its effects if actions remain passive. Distinct marine organisms occupying different niches could be used in Cartagena Bay to develop a biomonitoring program. The *Isognomon alatus* (filtering bivalve) and *Mugil incilis* (predator fish) could be included in ecotoxicological analyses to evaluate the disturbance of the ecosystem and to determine the negative impacts of multiple pollutants at the molecular, cellular, individual, population, and community levels, as well as the influence on the human health of surrounding communities.

## Figures and Tables

**Table 1:** Summary of DPSIR components and trends in Cartagena Bay.

DPSIR Component	Trends
Drivers	The population increases 1.16% per year [103]; almost 30% of the inhabitants live in poverty and 5.5% in extreme poverty [97]; concentration of high pollutant industries; weak land use policies and controls; an increase in tourism [75,104]; and a temperature increase of +0.9 to +2.23 °C, a precipitation decrease by 15% to 17%, a rise in the average sea level by +15 to +20 cm, and a 30% increase in the intensity of extreme precipitation to the year 2100, due to climate change scenarios [105,106].
Pressures	Informality and low adaptation of sustainable practices in economic activities; increased solid waste generation and wastewater discharges; increased water demand from tourism and industrial activities [75,104,107,108,109]; permanent sediment and pollutant loads from the Dique Channel [93,94]; the occurrence of extreme events [105,110]; and land use changes related to the loss of productive lands, filling of coasts, occupation of conservation areas, sediment loads, and coastal erosion [65,110,111,112,113,114].
State	Low environmental quality [115]; degraded ecosystems; the presence of persistent organic pollutants in sediments from different areas of the bay; metals As, Cd, Cr, Cu, Hg, and Pb at levels above the threshold effect level [80,116]; solid waste and the contamination of beaches [104,117,118]; threats to species of interest due to degradation of refuges and breeding areas and overfishing [119,120]; and a high vulnerability to global change, with scenarios directly compromising 27.5% of the population and a risk of flooding in 28% of industries and 35% of public infrastructure [121].
Impacts	Loss of habitat; seagrasses’ reduction by 63% in the last 25 years [66]; decrease in the coral community [64]; loss of mangroves; reduced connectivity between ecosystems [65,86]; alteration in the condition of fish related to increased infection by parasites [122]; bioaccumulation of organic contaminants and metals in different species [59,123,124,125]; increased environmental health threats for surrounding populations [81,82]; and the alteration of the physicochemical and microbiological water quality of the bay [115,126,127,128].
Responses	Education programs; strengthening pollution control and policies; the implementation of climate change adaptation programs; institutional articulation for environmental monitoring; and access to information systems [75,121,129].

**Table 2:** Individual PAH concentrations in sediments.

PAH Compound (ng g^−1^)	Study Area		
	Cartagena Bay (2003–2004)	Cartagena Bay (2017–2018)	Santa Marta Bay (2017–2018)
Acenaphthene		1.6	0
Acenaphthylene		5.8	0
Anthracene	37.5		
Benzo[a]anthracene	364.0	27.8	2.7
Benzo[a]pyrene	156.0	143.2	0
Benzo[b]flurantene	526.0	38.3	3.4
Benzo[g,h,i]perylene	145.0	27.0	1.9
Chrysene	252.0		
Dibenzo[a,h]anthracene	138.0		
Fluoranthene	68.4		
Fluorene	13.8	5.4	4.4
Indeno(1,2,3,cd)pyrene	36.3		
Naphthalene		2.3	1.9
Phenanthrene	105.0	46.7	11.4
Pyrene	250.0	29.0	4.7
Reference	[123]	[148]	

**Table 3:** Metal concentration in surface sediments from Cartagena Bay reported in different studies.

Metal (µg/g)			Reports of Trace Metals in Sediments (Year of Sampling)					Threshold Effect Level (TEL)
	2018	2015	2014–2015	2014	2012–2013	2006	1996	
As	3.62–20.6	4.1–13.1			2–8.5			7.24
Cd	0.11–2.1	0.2–2.3	0.232–0.877	0.015–0.057	0.13–0.55			0.68
Cr	24.1–268.2	22.6–137.2	5.9–59.8		5.1–18.7			52.3
Cu	11.5–147.7	20.5–429.0	3.1–38.6		6.8–65			18.7
Hg	0.01–0.84		0.065–0.30		0.02–0.17	0.02–0.55	0.094–10.29	0.13
Ni	11.2–67.1		24.6–32.7	14.9–23.9	3.9–11.3			15.9
Pb	3.6–54.4	7.7–37.1	1.6–14.6	1.4–2.0	2.7–6.4			30.24
Sn	0.1–3.3				0.20–0.53			0.048
Zn				46–78	28–34			124
Sampling sites	12	10	8	2	4	5	6	
Reference	[80]	[116]	[135]	[155]	[50]	[154]	[73]	[139]

**Table 4:** Trophic levels of marine organisms reported in different studies in Cartagena Bay. The data were obtained from the FAO Area, Exclusive Economic Zone (EEZ), and Large Marine Ecosystem (LME) datasets of Sea Around Us [158]. In addition, in cases without information in this database, other references were reviewed.

Species	Trophic Level	Data Base/Reference
*Triportheus magdalenae*	0.12	[159]
*Crassostrea rhizophora*	2.00	LME
*Saccostrea* sp.	2.00	FAO Area
*Mugil incilis*	2.01	LME
*Kyphosus* sp.	2.05	LME
*Stramonita haemastoma*	2.10	FAO Area
*Mugil cephalus*	2.13	LME
*Penaeus* *vannamei*	2.70	FAO Area
*Archosargus rhomboidalis*	2.89	EEZ
*Eugerres plumieri*	3.29	LME
*Gerres cinereus*	3.47	LME
*Elops saurus*	3.49	LME
*Bagre marinus*	3.51	EEZ
*Chloroscombrus chrysurus*	3.54	EEZ
*Dactylopterus volitans*	3.65	FAO Area
*Haemulon steindachneri*	3.73	LME
*Cathorops mapale*	3.77	[160]
*Lutjanus synagris*	3.82	EEZ
*Lutjanus* cf. *griseus*	3.90	[161]
*Callinectes sapidus*	4.00	LME
*Centropomus undecimalis*	4.17	EEZ
*Cynoscion jamaicensis*	4.20	LME
*Caranx hipos*	4.23	[160]
*Oligoplites saliens*	4.30	[162]
*Trichiurus lepturus*	4.42	EEZ
*Seriola rivoliana*	4.45	FAO Area
*Opisthonema oglinum*	4.50	EEZ
*Isognomon alatus*	No information	
*Callinectes bocourti*	No information	
*Sciades herzbergi*	No information	
*Donax denticulatus*	No information	

**Table 5:** Per- and polyfluoroalkyl substance (PFAS), polycyclic aromatic hydrocarbon (PAHs), and pesticide (ng/g) contents on marine organisms found in Cartagena Bay.

Sampling Season	Species	Taxonomic Group	Pollutant Concentration (ng/g)	Trophic Level	Reference
December 2003	*Mugil incilis*	Fish	PFOA: 370 ± 65.7 PFHxS: 0.489 ± 0.08 PFOSA: <0.3	Detritivorous	[163]
August 2003 to June 2004	*Mugil incilis*	Fish	∑OH-PAH: 1250	Detritivorous	[123]
January, June, and November 2008	*Penaeus vannamei*	Crustacean	Metoxychlor: 94.6–163 Endrinsulfate: 1.6–17.9 BHC: 9.4–15.1 Endrinaldehyde: 3.4–5.6	Detritivorous	[124]
June–November 2009	*Mugil incilis*	Fish	β-HCH: 0.00185–0.00638	Detritivorous	[136]
			Aldrin: 0.00115–0.00333		
			4,4′-DDD: 0.00404–0.00452		
			γ-HCH: 0.00851 ± 0.002		
			Heptachlor: 0.00436–0.00725		
			Endosulfan: 0.00415 ± 0.001		
			4,4′-DDE: 0.00401 ± 0.001		
			Dieldrin: 0.00206 ± 0.000		
October 2012 and March 2013	*Crassostrea rhizophora*	Bivalve	ΣPAHs: 41.0–1299.5 ΣHMWPAHs: 87.8–986.3 ΣLMWPAHs: 0.8–265.6 Galaxolide (HHCB): 0.4–71.0 Tonalide (AHTN): 0.2–48.7 ΣMusks: 0.4–119.6 ΣPCBs (PCB_7_): 0.0–29.3 ΣPOPs: 6.1–140.6	Filter-feeding	[164]
October 2012 March 2013	*Saccostrea* sp.	Bivalve	HCHs: <LOD 50 DDT: <LOD 2 Chlorpyrifos:<LOD 2	Filter-feeding	[50]

**Table 6:** Metal (µg/g dw) contents on marine organisms found in Cartagena.

Sampling Season	Species	Taxonomic Group	Metal Concentration	Trophic Level	Reference
November 1980	*Crassotrea rhizophorae* *Isognomon alatus*	Bivalve	Cd: 2.51–15.9 0.80–15.60 Cu: 11.70–23 0.87–4.77 Pb: 1.26–5.13 0.75–3.16	Filter-feeding	[165]
March, May, August, and November 1996	*Mugil incilis* *Eugerres plumieri*	Fish	Hg: 0.007 to 0.166 0.019 to 0.852	Detritivorous Omnivorous	[73]
March–April, May–June July–August 2007	*Not reported*	Bivalve	Cd: 4.98 to 21.33	Filter-feeding	[137]
2004–2005	*Callinectes sapidus* *Callinectes bocourti*	Crustacean	Hg: 0.124 ± 0.011	Omnivorous	[125]
March–July 2006	*Chloroscombrus chrysurus* *Cynoscion jamaicensis* *Caranx hipos* *Elops saurus* *Lutjanus synagris* *Centropomus undecimalis* *Trichiurus lepturus*	Fish	Hg: 0.26 ± 0.16 0.11 ± 0.05 0.09 ± 0.03 0.05 ± 0.02 0.08 ± 0.01 0.09 ± 0.04 0.08 ± 0.03	Carnivorous Second Order	[59]
	*Opisthonema oglinum* *Dactylopterus volitans* *Gerres cinereus* *Eugerres plumieri* *Haemulon steindachneri* *Oligoplites saliens* *Sciades herzbergi*	Fish	Hg: 0.11 ± 0.04 0.05 ± 0.02 0.10 ± 0.08 0.04 ± 0.04 0.08 ± 0.04 0.09 ± 0.02 0.11 ± 0.06	Carnivorous Third Order	
	*Triportheus magdalenae* *Archosargus rhomboidalis*	Fish	Hg: 0.07 + 0.01	Omnivorous	
	*Mugil cephalus* *Mugil incilis*	Fish	Hg: 0.02 ± 0.01 0.03 ± 0.02	Detritivorous	
2013	*Stramonita haemastoma*	Gastropod	As: 0.158 Cd: 0.02 Cr: 0.056 Cu: 0.880 Ni: <0.01 Pb: 0.695 Sn: 0.126 Zn: 0.479	Detritivorous	[166]
September 2012 and May 2013	*Donax denticulatus*	Bivalve	Cd: 0.040 Hg: 0.006 Pb: 0.060	Filter-feeding	[167]
October 2012 and March 2013	*Crassostrea rhizophora*	Bivalve	ΣAg, Al, As, Cd, Cr, Cu, Hg, Ni, Pb, Ti, V, and Zn 629.80–2490.53	Filter-feeding	[164]
October 2012 and March 2013	*Saccostrea* sp.	Bivalve	As: 5.96–7.62 Cd: 3.43–15.88 Cr: 0.23–9.14 Cu: 38.72–296.68 Hg: 0.04–0.09 Pb: 0.15–0.75 Ni: 0.43–1.61 Sn: 0–1.05 Zn: 488.6–3390.2	Filter-feeding	[50]
June–July 2014	*Kyphosus* sp. *Seriola rivoliana* *Lutjanus* cf. *griseus* *Mugil incilis* *Cathorops mapale* *Bagre marinus.*	Fish	Zn: 0.330–3.90 Cd: ND-0.0053 Ni: ND-0.500 Pb: 0.010–0.110	Carnivorous	[155]

**Table 7:** Sentinel species and biomarkers employed in the biomonitoring of Cartagena Bay.

Species	Biomarker Level	Method	Inference	Reference
*Mugil incilis*	Morphology	Measurements of total length and weight; condition factor; gill-somatic index; hepato-somatic index; spleen-somatic index	*t*-test between sampling sites.	[163]
	Morphology	Measurements of total length and weight; condition factor; hepato-somatic index; bazosomatic index	Correlation of morphometric parameters, parasitic intensity, and concentration of organochlorine pesticides and comparison with histopathological changes	[136]
	Histology	Parasitic infection, histopathology recorded by lesions, nonspecific inflammatory changes (infiltration of inflammatory cells and granulomatosis), necrosis, apoptosis, and the presence of melano-macrophage centers (MMCs)		
	Molecular	RNA-Seq gene markers of heavy metal exposure, xenobiotic metabolism, nuclear receptor modulation, oxidative stress, DNA damage, inflammation, and lipid metabolism	Gene expression	[58]
18 Fish species	Morphology	Measurements of total length and weight; condition factor; gill-somatic index; hepato-somatic index; spleen-somatic index	Spearman correlations between T-Hg levels and morphometric indexes	[59]
*Crassostrea rhizophorae*	Histology	Parasitic infection, histopathology with inflammatory response index (IRI), haemocytic infiltration, brown cell aggregates, and disseminated neoplasia	Statistical differences between sampling sites and season.	[168]
	Morphology	Flesh condition index, shell length, flesh dry weight, shell cavity volume, gamete developmental stage		
	Molecular	Total metallothionein proteins, cholinesterase activity (ChE), eserine-resistant cholinesterase (Er-ChE) activity in digestive glands and gills	Statistical differences between sampling sites.	[50]
*Stramonita haemastoma*	Morphology	Imposex: relative penis length index (RPLI), relative penis size index (RPSI).	Prevalence by sampling sites.	[166]
*Donax denticulatus*	Morphology	Measurements of anteroposterior length, total width, total height, total weight, and tissue biomass	Pearson correlation for Hg, Pb, and Cd (not significative) and distribution of sampling sites according to Principal Components Analysis.	[167]

## Data Availability

Data sharing is not applicable to this article.
